# A reconfigurable intelligent surface with integrated sensing capability

**DOI:** 10.1038/s41598-021-99722-x

**Published:** 2021-10-20

**Authors:** Idban Alamzadeh, George C. Alexandropoulos, Nir Shlezinger, Mohammadreza F. Imani

**Affiliations:** 1 Department of Electrical, Computer and Energy Engineering, Arizona State University, Tempe, AZ 85281 USA; 2 Department of Informatics and Telecommunications, National and Kapodistrian University of Athens, 15784 Athens, Greece; 3 School of Electrical and Computer Engineering, Ben-Gurion University of the Negev, Be’er-Sheva, Israel

**Keywords:** Electrical and electronic engineering, Information theory and computation, Electronic properties and materials, Magnetic properties and materials, Metamaterials

## Abstract

Reconfigurable reflective surfaces can alter the propagation environment to improve wireless communication and power transfer. Paramount to this operation—which has attracted much attention recently—is the assumption that the reflective surface has prior knowledge of the propagation environment, for example, the direction/location of the transmitter and the intended receiver(s). To address this need, we propose a reconfigurable reflective metasurface with integrated sensing capabilities. By modifying the tunable meta-atoms constituting the metasurface, we couple small portions of the incident wave to an array of sensing waveguides. As an illustrative example, we demonstrate the ability to use the sampled incident wave to detect its angle of arrival. In addition, we propose and numerically demonstrate the possibility to reduce the required sensors, i.e., the number of radio frequency (RF) chains needed to acquire the sensed signals, by leveraging the inherent metasurface’s tunable multiplexing capability. A reconfigurable reflective metasurface with integrated sensing capabilities can benefit wireless communications, wireless power transfer, RF sensing, and smart sensors.

## Introduction

Reconfigurable intelligent surfaces (RISs)—also known as intelligent reflecting surfaces—have garnered much attention recently^1–16^. Much of this attention stems from their recent revolutionary applications in wireless communications where they have shown the possibility to engineer wireless communication environment, rendering the communication channel into a design knob^17,18^. For example, blockage of the waves by obstacles or their diffraction from the surfaces of the objects in a typical propagation environment may significantly reduce the signal strength and consequently, yield lower data rate at the intended receiver(s). Deploying RISs has the potential to mitigate this signal squandering by redirecting the blocked or diffracted wave in a passive manner toward the intended receiver(s). To accomplish the intelligent engineering of the propagation environment, *knowledge* of the instantaneous channel/environment state at the constitutive elements of the RIS is necessary so that the reflection patterns of the RIS are set to redirect the signal towards the intended targets. In other words, without the knowledge of the propagation environment at each constitutive element of the RISs, the full potentials of RISs cannot be realized.

To address the aforementioned need, some recent works have relied on estimating the cascaded channel describing the end-to-end transmitter-RIS-receiver link^19–23^. However, this approach may require costly estimation overhead and impose restrictions on various network management mechanisms, as it cannot decouple the transmitter-RIS and the RIS-receiver channels. Alternatively, it has been suggested to equip RISs with dedicated receiving antennas and RF chains that can be used to estimate the wireless environment at the RIS^24–26^. This approach, however, might require more than few RF chains when the number of RIS elements increases significantly. Furthermore, since a portion of the RISs surface is dedicated to receiving, the overall effective surface of the RIS is reduced. A different approach was proposed in^27^ where the RIS’s elements were envisioned to be connected to a *communication nano-network*. The RIS’s elements are switched between different states till the power sensed by the nano-network is maximized. Comparing the states of the elements with a look-up table, one can thus implicitly estimate the channel from the incident wave. This method, referred to as *ABSense*, suffers from limited spatial resolution, and relies on a specific type of RISs referred to as *HyperSurfaces*, which is a technology in relatively early stages of development and a fully-functional one with integrated sensing capability has not been reported yet^28,29^. Another solution proposed to the sensing challenge is to use optical or infrared (IR) images in parallel with the RIS. This method, which requires supporting imaging technologies, especially works well for direct line-of-sight (LOS) communication scenarios^30^, but cannot be easily extended to non-line-of-sight or low ambient light scenarios.

In this work, we propose an alternative solution for the RIS network incorporation problem by designing a reconfigurable reflective metasurface configuration with *integrated sensing capability*. Towards this goal, we modify the reconfigurable meta-atoms constituting the metasurface such that a portion of the incident wave couples to a waveguide where it can be sampled by a receiving circuitry. Using the sampled signal, one can deduce necessary information about the propagation environment. As a demonstrative example of the proposed RIS’s design and functionality, we show that we can estimate the angle of arrival (AoA) of an incident signal. A key challenge in implementing such RISs at a large scale follows from the fact that sensing at each meta-atom is likely to lead to a prohibitively large number of receiving RF chains. On the other hand, maintaining sufficient spatial resolution is a necessity for robust channel estimation. To tackle this problem, we use an approach reminiscent of compressed sensing techniques^31^ to reduce the number of required RF chains. Specifically, we leverage the reconfigurable metasurface aperture and the inherent coupling between its elements. The scattering of the incident wave inside the metasurface and the coupling between elements effectively multiplexes the field incident on each element^32–34^. Consequently, the received signals are obtained via a form of hybrid analog/digital processing, where the field received by each RF chain has information about the incident wave on all elements. By reconfiguring the metasurface aperture randomly, we can thus generate diverse sets of measurements from the incident waves, even when using few RF chains. This operation has been used before for computational microwave imaging to reduce the required number of receivers^35^ and is adapted here to reduce the number of receiving circuitry for retrieval of channel information. In a similar fashion, approaches to estimate large channels with reduced number of RF channels are of particular importance in millimeter wave multiple input multiple output (MIMO) systems with hybrid analog/digital beamforming^36^.

It is worth noting that the proposed integrated sensing operation is distinct from recent works on using RISs for localizing users^37,38^ and sensing/imaging objects^39–43^. In these previous works, the ability of the RIS to generate distinct (reflected) patterns is used to probe the scene in front of the RIS, for example, to image an object of interest or even deduce human gesture. The receiver is usually a dedicated antenna located near the RIS. This setup closely resembles the configuration of single-pixel imaging/sensing pursued in optics^44^. While such techniques provide some useful information about the propagation environment (e.g. where a receiver in LOS is located), they are very distinct from our proposed operation, which allows for obtaining information about the channel state at each meta-atom. More importantly, methods in^39–43^ only work when there is a direct path between the transmitters, RIS, and receivers, and the pattern generated by the RIS is known (which requires minimal change to the environment). Our proposed operation is more suitable for the general case where one cannot make such assumptions about the locations of the transmitters and receivers, or the environment. More recently, integrated sensing capabilities for hybrid reflecting/receiving metasurfaces was suggested in^45,46^; however, the main focus of the numerical study in^45,46^ was on the communication aspect of the hybrid configuration. This paper, in contrast, focuses on the hardware aspect of such hybrid RISs and explicates the design considerations of a metasurface with integrated sensing capabilities.

This paper is organized as follows. First, the design of a meta-atom with integrated sensing capability is presented. We then examine a 1D array of such meta-atoms in full-wave simulations and demonstrate that the sampled signal contains information about the channel. As an example, we use the sampled signal to estimate the AoA of the incident wave. Next, we show that we can reduce the number of receiving circuitry by leveraging the metasurface reconfigurable patterns. Lastly, we present preliminary results on the proposed RIS’s capability to redirect the reflected beam towards prescribed directions—as desired for RIS-empowered wireless communication systems.

## Proposed design and operation

### Metamaterial element design

**Figure 1:**
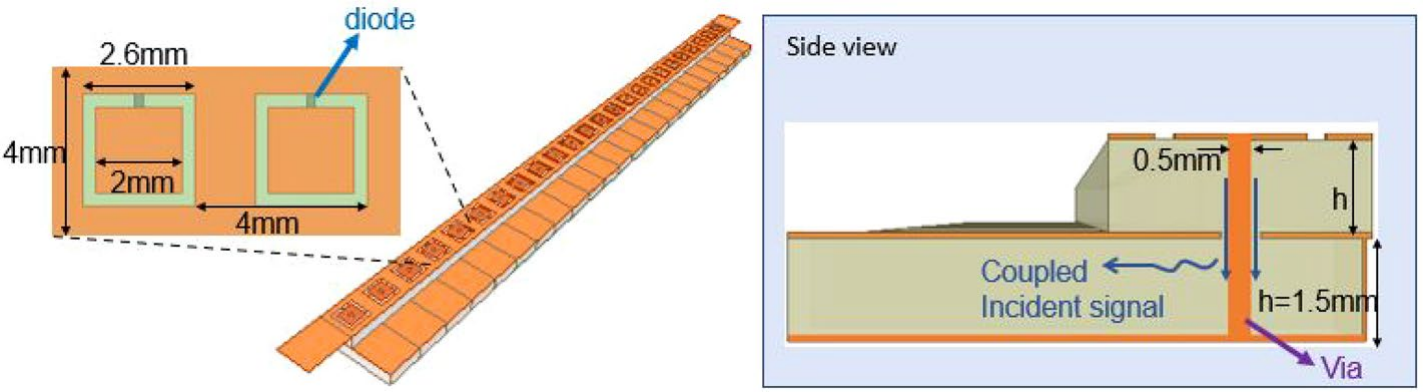
Proposed hybrid RIS hardware design with integrated sensing capability.

The proposed metasurface is shown schematically in Fig. 1. It consists of well-known mushroom structures^47^, each loaded with a varactor diode. This reconfigurable capacitance results in a reconfigurable resonance frequency, and consequently, a reconfigurable effective impedance. We have selected this element since it provides a simple mechanism to realize high reflectivity with reconfigurable phases. However, the proposed operation is not unique to this geometry and can easily be used in other configurations such as metallic patches^48^, complimentary electric-inductive-capacitive (cELCs)^49^, etc. To address each meta-atom independently, as required for forming desired reflection patterns, the via of the mushroom structure extends through the bottom conductive plate (similar to the structure in^50^). An annular slot separates the via from the ground plane beneath the substrate. This annular slot allows for coupling of the incident wave to another layer. In previous works, an RF choke (in the form of a radial stub) was placed at the end of this via to diminish RF coupling^51^. In this paper, we instead take advantage of this annular slot to sense the incident wave (see Fig. 1).

**Figure 2:**
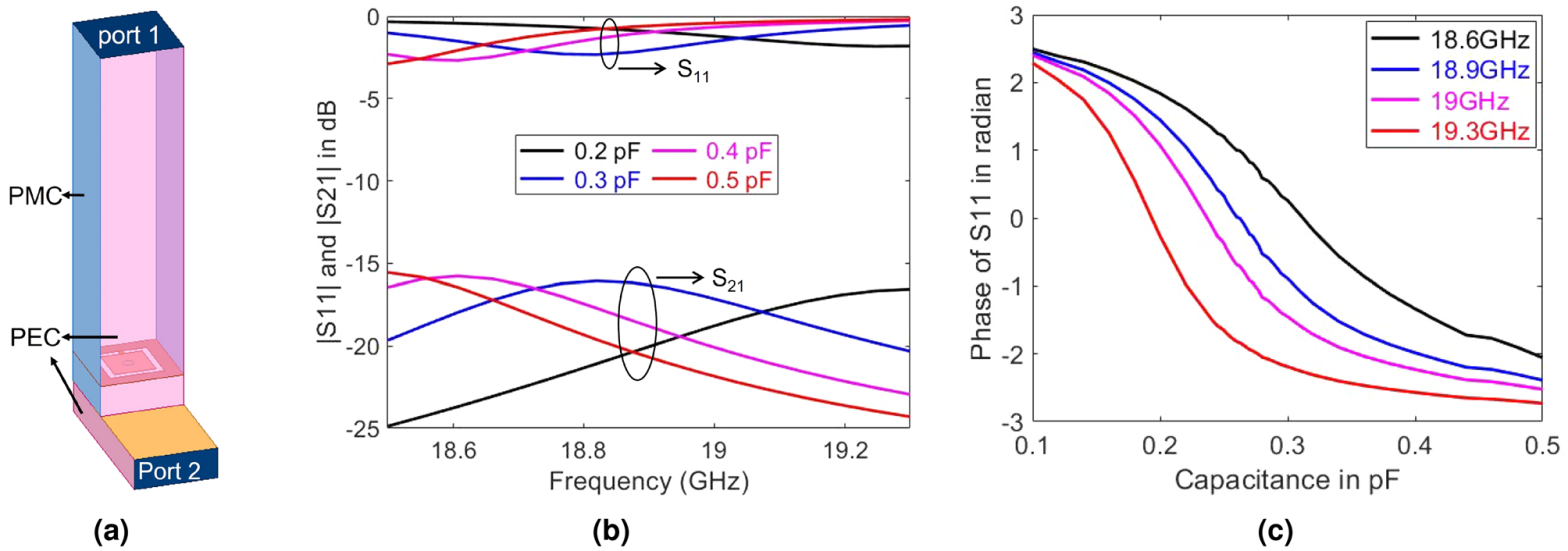
(**a**) The schematic of the setup used for simulating a single hybrid meta-atom. PEC and PMC refer respectively to perfect electric conductor and perfect magnetic conductor. (**b**) Simulated reflection, $|S_{11}|$, and coupling, $|S_{21}|$, coefficients as a function of frequency. (**c**) Phase of $S_{11}$ vs. capacitance at several frequencies for a meta-atom with integrated sensing capability.

To design this element, we have simulated it in Ansys HFSS in the setup shown in Fig. 2a. In the simulations, the dielectric substrate of the meta-atom is assumed to be RO4003 with relative permittivity of 3.55 and loss tangent of 0.0027. In our studies, we do not include the circuitry to change the DC voltage and model the varactor as a lumped element with a variable capacitor. The design parameters were chosen for operation at around 19 GHz (similar to that of^50^). Nonetheless, the proposed design and operation can be easily extended to other frequency ranges. The important geometrical dimensions of the proposed hardware design for operation at around 19 GHz are shown in Fig. 1. The element size and the spacing between elements are around a quarter of the wavelength. Using subwavelength elements allows for better approximations of the necessary phase profile (or hologram) to redirect the reflected beam toward desired directions^52^. Utilizing even smaller element size and spacing may in fact improve beam redirecting performance at the cost of large inter-element coupling and costly fabrication. Larger element size and spacing are usually associated with lower performance in realizing desired beams. Hence, quarter wavelength element size seems to be a practical middle ground. Note that from the sensing perspective, elements separated by no more than half wavelength should theoretically suffice.

The primary criterion in designing the proposed meta-atom is to ensure a small portion of the incident wave can be sensed while maintaining sufficient reflective power and a reconfigurable reflective phase (to form desired reflection patterns). To produce the desired reconfigurable reflection, we realize a resonance frequency in the band of operation and change it by altering the varactor capacitance. To control the amount of coupling, we can adjust the diameter of the annular slot and the characteristic dimensions of the coupling waveguides. Here, we select substrate integrated waveguides (SIWs) for guiding the sampled signal for multiple reasons. In contrast to microstrips or similar geometries that can couple to each other, SIWs confine electromagnetic waves within their boundaries. Besides, the junction of the via and microstrips can result in spurious radiation, which would be detrimental for our proposed operation. Lastly, one can further tailor the level of coupling between the SIW and the meta-atom by changing the cutoff frequency of the SIW and/or by tweaking the distance from the via to the sides of the SIW.

Using the simulation setup shown in Fig. 2a, we can examine both the reflection from the meta-atom and the coupling to the SIW using the scattering parameters. Specifically, $S_{11}$ represents the reflection from the element while $S_{21}$ measures the signal coupled to the sampling SIW. The simulated scattering parameters of the designed meta-atom element are shown in Fig. 2. Note that we de-embedded port 1 to the surface of the metasurface to remove excessive phase delay caused by propagation between port 1 and the element. In these simulations, the diameter of the via and the annular slot are respectively 0.5 mm and 0.6 mm, the SIW dimensions are $8.7 \;\mathrm {mm} \times 4 \;\mathrm {mm} \times 1.5 \;\mathrm {mm}$, and the distance between the via and the shorted edge of the SIW is $1.6 \;\mathrm {mm}$. The via is equidistant from the other two side walls of the SIW. It is evident from Fig. 2 that the coupled signal, $|S_{21}|$, is below $-15$ dB for the whole frequency range of operation while the magnitude of the reflection coefficient, $|S_{11}|$, is above $-2.5$ dB. This trend is maintained over the selected capacitor range as depicted by some illustrative examples in Fig. 2. The phase responses of $S_{11}$ in Fig. 2c portray the variation of reflection phases as the effective capacitance of the varactor changes—a behavior required at the surface of a RIS to redirect the reflected beam towards a desired angle.

The scattering parameters presented in Fig. 2b,c confirm that the proposed design satisfies our primary design criterion. In the remainder of this paper, we focus on demonstrating the hardware feasibility of the proposed hybrid RIS operation. It is important to note that the optimal ratio between reflected and sensed signals needs to be established for accurate channel estimation while successfully reflecting from RIS to desired targets. A preliminary investigation into this trade-off has been reported in^45,46^. Those works, however, utilized a simplified model for the proposed RIS. A more comprehensive investigation is needed with a physically accurate model of the RIS meta-atom. Furthermore, those studies did not examine the case of a reconfigurable sensing matrix as we will later demonstrate in this work. A more in-depth analysis to achieve optimal reflection and receive characteristics is left for future works. The studies conducted in this paper paves the way for such investigations.

### Sensing capability

**Figure 3:**
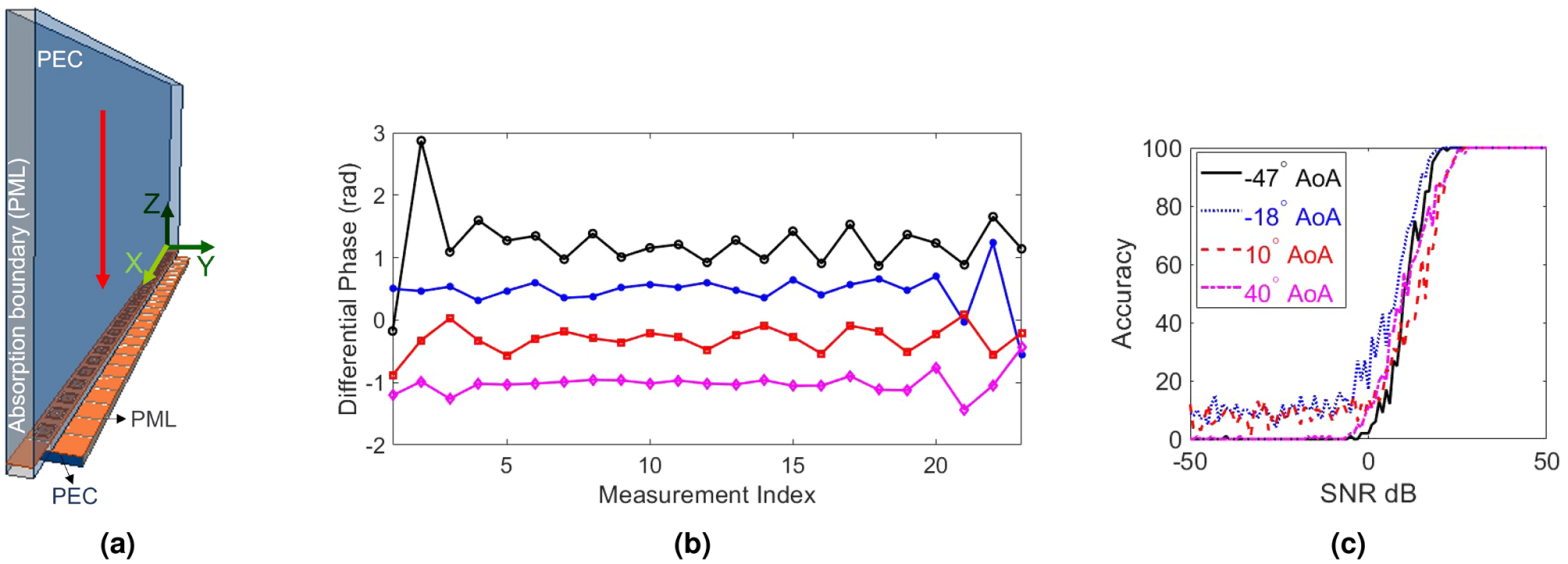
(**a**) The setup for illuminating the RIS with a Gaussian beam. The red arrow indicates the normal incidence. PML refer to perfectly matched layer. (**b**) Differential phase along the 1D metasurface array for four different incident angles captured inside SIWs. (**c**) AoA estimation accuracy (in percentage) as a function of the signal-to-noise ratio (SNR). The incident angles are the same in both plots.

To demonstrate the possibility to deduce information about the environment using the proposed design depicted in Fig. 1, we have simulated a 1D array (for simplicity) of 24 hybrid meta-elements. The setup for this simulation is shown in Fig. 3a. In our studies, we have considered that the 1D array is periodic along the *Y* axis. Our intention is to show that our proposed structure can infer the direction of the incident beams and steer them in the *XZ*-plane. Extending these capabilities to full space is a subject of future work. We assume the varactor attached to each element has an effective capacitance of 0.4 pF. A 36 mm wide Gaussian beam at 18.9 GHz is chosen as the incident wave. The phase differences measured at the SIWs for different incident angles (AoAs) are shown in Fig. 3b. In this figure, we report the phase differences between adjacent elements since the absolute phases are reference-dependent and are not meaningful. For example, the measurement indexed 7 is the difference between the phases of the fields sampled by the $7\hbox {th}$ and $8\hbox {th}$ SIWs. By examining Fig. 3, we clearly see that the measured phase difference changes as a function of AoA. We also see that the phase difference is not constant across all the elements for the same incident angle. This is because of the coupling between meta-atoms and the scattering of the waves inside the substrate below the mushroom structures.

### AoA estimation

The phase differences sampled along the metasurface (shown in Fig. 3b), despite showing unique dependency on the AoA, do not exhibit a one-to-one relationship. For example, we cannot equate the (average) phase differences measured to the differences in the AoAs. Furthermore, the field scattering inside the substrate beneath the mushroom structures as well as the inter-element coupling further complicates this relationship. To estimate the AOA of the impinging signal, we propose an intuitive process based on the comparison between the differential phase for an unknown incident beam with a set of pre-recorded measurements. To this end, we have simulated the structure for a set of 26 AoAs between $-57^{\circ }$ and $57^{\circ }$. The resulting phase difference is recorded in a sensing matrix, $\mathbf {H}$, whose (*i*, *j*) element (i.e. $H_{ij}$) corresponds to the measured phase difference between the $i\hbox {th}$ and the $(i+1)\hbox {th}$ waveguides for the $j\hbox {th}$ incident angle. Thus, $\mathbf {H}$ is an $M \times N$ real-valued matrix corresponding to $(M+1)$ sensed samples and *N* AoAs ($N=26$ for the results reported in Fig. 3c). The $M\times 1$ simulated phase difference vector $\mathbf {g}$ for an unknown AoA is then weighted by $\mathbf {H}$.

1 $$\begin{aligned} \mathbf {w} = \mathbf {H^{'} g}. \end{aligned}$$

where $'$ denotes transpose. The column of $\mathbf {H}^{'}\mathbf {H}$ which has the smallest distance from $\mathbf {w}$ thus corresponds to the estimated AoA. Mathematically, if we assume $\mathbf {h}_j$ is the *j*th column of $\mathbf {H'H}$, the column that exhibits the minimum distance, $J_{est}$, is given by:

2 $$\begin{aligned} {J}_{est} = \text {arg}\min _{j=1\dots N} \Vert \mathbf {w} - \mathbf {h}_j \Vert _{2}, \end{aligned}$$

where $\Vert .\Vert _2$ is the Euclidean norm. Once we know $J_{est}$, we can use our reference AoAs in the sensing matrix to estimate the unknown AoA. The accuracy of this approach in determining the actual AoA is thus limited by the resolution of the reference AoAs used in populating $\mathbf {H}$ (in the numerical studies presented have used a phase resolution of $4.6^{\circ }$). We thus define our estimation accuracy, $\eta$, in a binary fashion. When the difference between the estimated AoA and the actual test AoA is less than $2.4^{\circ }$ (slightly larger than half of the dictionary resolution, $4.6^{\circ }$, to accommodate cases midway between two values in the dictionary), the estimation is considered to be accurate and we assign $\eta =1$. If the estimated AoA is out of the resolution around the incident AoA, we consider that a failure and assign $\eta =0$.

To test the process described above, we simulate this setup with four test beams incident at angles that are not in our dictionary. These angles are selected to be distributed over the whole range of interest $(-57^\circ$ to $57^\circ )$. We will use these test AoAs for all the studies reported in this paper. For each test incident angle, we also study the effect of the system’s noise on the estimation accuracy by adding additive white Gaussian noise to the sampled signals (using the built-in awgn function in MATLAB). For a given signal-to-noise ratio (SNR) level, we evaluate $\eta$ by averaging over 100 noise realizations. The results of our AoA estimation method for different incident angles are shown in Fig. 3c. It is observed that we can recover the AoA within the resolution of our dictionary, thereby verifying the proposed design and operation. It is worth emphasizing that the angular selectivity of this configuration is a function of various factors, including noise, retrieval algorithm, incident beam profile, resolution of the sensing matrix, relative direction of the incident beam to the norm and/or to the angles in sensing matrix, the overall size of the RIS, and the number of sampling SIWs. Thoroughly characterizing these relationships is beyond the scope of this paper and is left for future works.

## Sparse sampling

**Figure 4:**
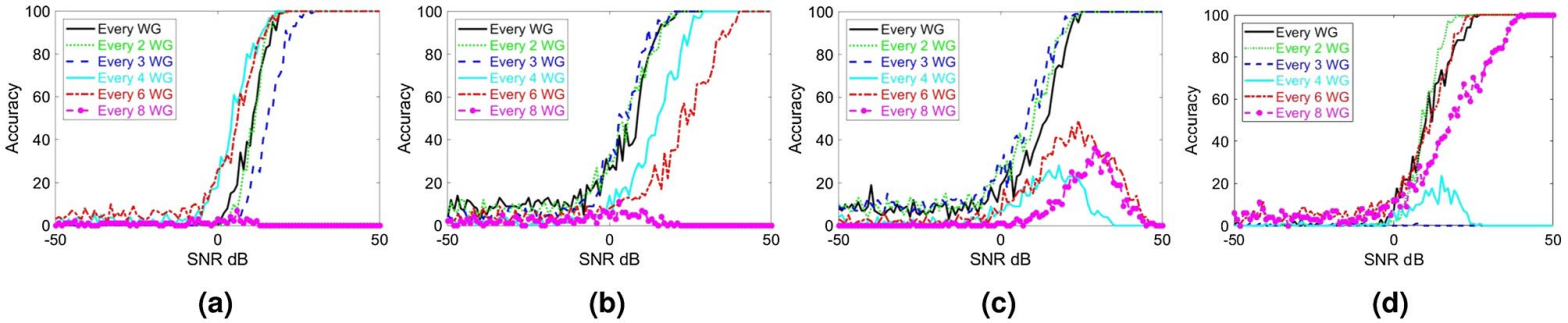
AoA estimation accuracy (in percentage) for wave incident at (**a**) $-47^{\circ }$, (**b**) $-18^{\circ }$, (**c**) $10^{\circ }$, and (**d**) $40^{\circ }$, calculated with different numbers of uniformly spaced waveguides (WG).

As noted earlier, the proposed RIS with integrated sensing capabilities may be infeasible to implement if all meta-atoms are connected to a receiving circuitry. As a result, it is vital to reduce the number of required receiving circuitries or receiving RF chains. In this section, we examine the impact of reducing the number of RF chains on the AoA estimation accuracy and propose a solution such that the reduction in receiving RF chains has minimal impact on estimation fidelity comparable to the accuracy plots in Fig. 3. Toward this goal, we first gradually decreased the number of uniformly spaced sampling RF chains and calculated the AoA estimation accuracy as a function of SNR. To evaluate this setup, we process the received signal from a subset of the waveguides and ignore the remaining ones. We start by selecting uniformly spaced subsets of the sampled signals, the results of which are reported in Fig. 4. It can be seen that reducing the number of measurements (or sensing circuits) degrades the fidelity in detecting the AoA. Interestingly, we can observe that the deterioration in the sensing capability is not the same for all the test angles since the sensing capability is a complicated function of multiple factors (including the number of SIWs), as discussed earlier. Nonetheless, a practically useful RIS needs to exhibit satisfactory accuracy in detecting all possible AoAs.

**Figure 5:**
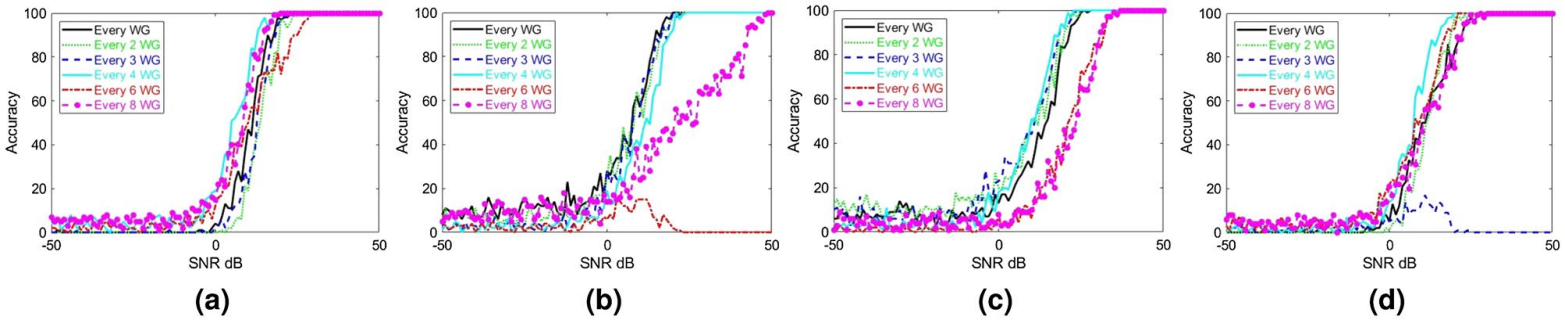
AoA estimation accuracy (in percentage) for wave incident at (**a**) $-47^{\circ }$, (**b**) $-18^{\circ }$, (**c**) $10^{\circ }$, and (**d**) $40^{\circ }$, calculated with different numbers of nonuniformly spaced waveguides (WG).

In order to facilitate sensing with reduced number of RF chains, we recall that the wave incident on the metasurface scatters inside the substrate beneath the mushroom structures. This results in a multiplexing effect where the field sampled at each SIW carries information about the field incident on all elements. This operation in fact shares similarities with previous works where the random multiplexing of information via a metasurface was used to recover a (sparse) scene information using measurements from few receivers^35^. To implement random multiplexing of information, we first examine non-uniform SIW distributions as we decreased the number of waveguides. Specifically, we slightly perturbed the uniform sampling distributions used in Fig. 4. The estimated accuracy when using such *nonuniform* sampling distributions are shown in Fig. 5. By comparing the results in Figs. 4 and 5, we see tangible improvement in the fidelity when using nonuniform sampling. In fact, similar results have also been reported when using a random distribution of metamaterial elements in previous works^35,53^ for computational microwave imaging. This observation also suggests that there can be an optimum distribution that may guarantee the desired level of accuracy. Examining such a possibility is left for future works.

In this work, we pursue a more pragmatic approach to maintaining high accuracy as we decrease the number of measurements: we randomize the effective capacitances loading the meta-atoms (referred to as *mask*s for brevity) to alter the random multiplexing inside the substrate. To demonstrate this process, we have simulated the sensing matrix and the test incident beams using masks with random distribution of 0.1 pF and 0.4 pF capacitances—in contrast to previous results which were all with uniform 0.4 pF capacitances. The measurements for each mask are concatenated to form a new sensing matrix where $(i,j)\hbox {th}$ entry corresponds to the $i\hbox {th}$ entry of the concatenated array of the differential phase for the $j\hbox {th}$ incident angle. Note that the same random masks are used for both populating the sensing matrix as well as when examining test incident beams. This operation also assumes that the location of a dynamic target is stationary during data acquisitions. This assumption is reasonable since varactor diodes can be tuned at a much faster rate than typical movements in a propagation environment.

**Figure 6:**
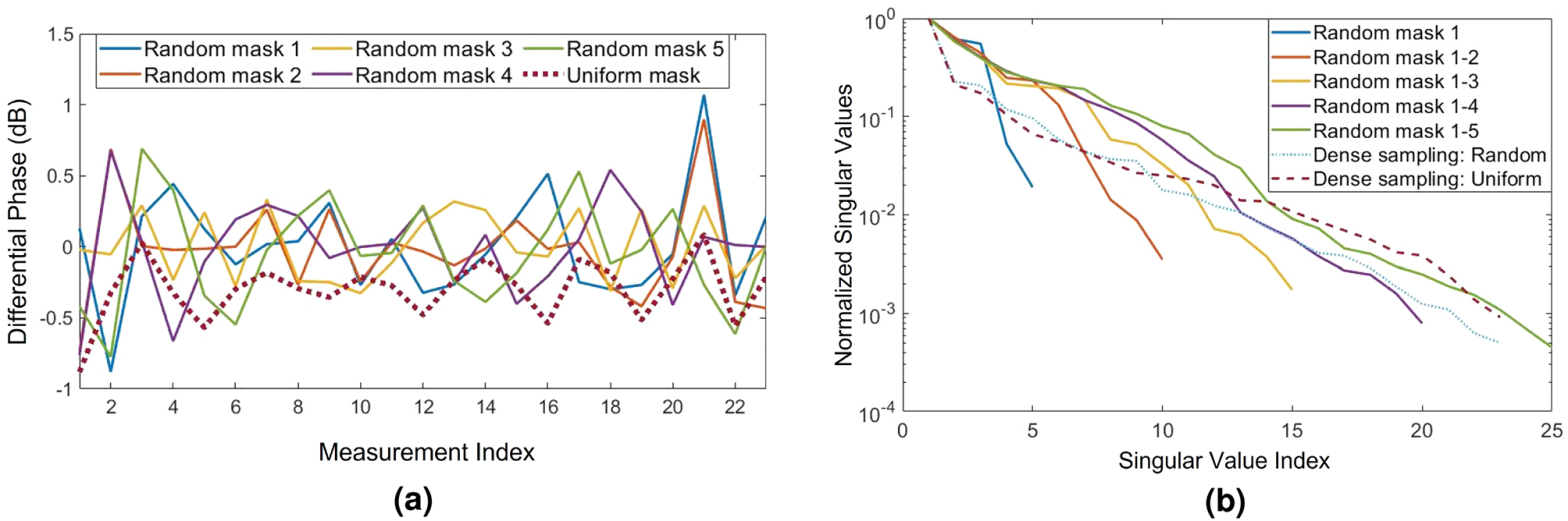
(**a**) Differential phase for uniform and different random capacitor combinations (masks) for $10^{\circ }$ incident angle. (**b**) Normalized singular values of the sensing matrix for a sparse sampling metasurface (with different numbers of random masks) in comparison to densely sampled uniform (and random) masks.

To visualize the impact of using random masks, we plot the differential phase for an example incident angle for different masks in Fig. 6a. As it can be seen, the differential phases change significantly as a result of changing the varactors capacitance distribution. Interestingly, we also observe a less uniform graph compared to the uniform mask, which implies we have further multiplexed the wave sampled by the waveguides. To illustrate the fact that using random masks yields new degrees of freedom for multiplexing, we have computed the singular value decomposition (SVD) of the sensing matrix as we add new masks (see Fig. 6b). In our numerical study, we keep the number of SIWs at 6 non-uniformly distributed ones (resulting in 5 differential phase values for each mask). It is clearly noted that the effective ranks of the sensing matrix (defined as the number of normalized singular values above a certain threshold) are increasing as we add new measurements via new masks. More specifically, we can make the following observations: 1) Since the slope of the singular value (or the rate it decreases) improves as we increase the number of masks, we can conclude that the new masks carry new information. 2) If we use a fixed mask—either uniform or random capacitance distribution— with all the 24 waveguide measurements (dotted-blue and dashed-magenta lines in Fig. 6b), we end up with almost similar condition numbers (defined as the ratio of maximum to minimum singular values) as in the case when we use sparse sampling but change the masks (green line in Fig. 6b). In other words, using random masks can allow us to reduce the number of SIWs without losing much information.

**Figure 7:**
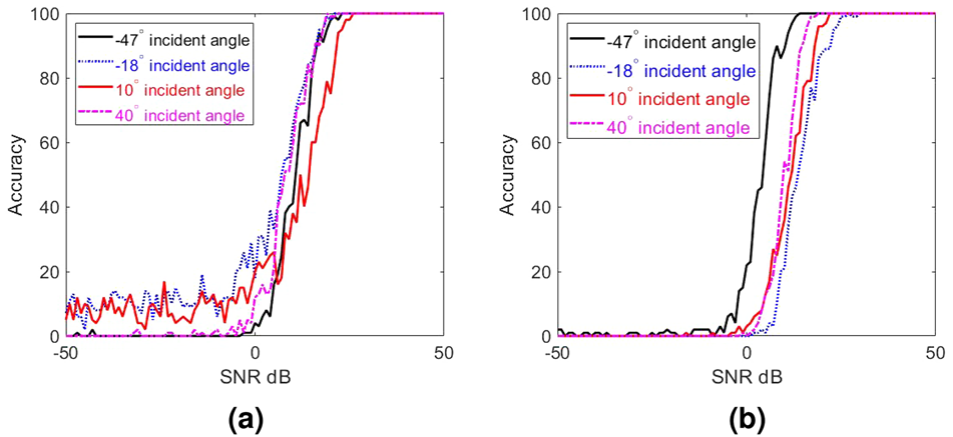
AoA estimation accuracy (in percentage) with incident signal sampled from a) all 24 waveguides with a uniform mask (**a** replica of the estimation accuracy presented on Fig. 3) and** b**) 6 waveguides with five random masks. The same set of AoAs is used in both cases.

To demonstrate the efficacy of the sparse sampling, the estimation accuracy for the same sets of test beams used in Fig. 3 but with signals sampled at only six SIWs (yielding total of five measurements per mask) is calculated. In these calculations, we have used five random masks. The results are presented in Fig. 7. Evidently, we can achieve the same level of accuracy as in the case of uniform dense sampling with received signals from only six SIWs if we use random masks. This means we can reduce the number of sampling RF chains by at least a factor of four. In future works, we will examine the possibility to even further reduce the number of required RF chains by, e.g., combining the signals sensed by multiple elements into a single RF chain, as proposed in^26^.

## Sculpting reflection patterns

For the proposed hybrid sensing/reflecting metasurface to be useful in RIS-empowered wireless communications, it also needs to redirect incident beams to intended users (in addition to its sensing capability). It is worth emphasizing that the proposed RIS configuration is not optimized for beam generation and our primary focus here has been on the design of an RIS that realizes simultaneous reflection and sensing of the incident wave. Nonetheless, it is important to show that the proposed RIS can still redirect reflected beams toward desired directions.

The physical basis for realizing the desired anomalous reflection is to form a phase gradient along the metasurface^54^. In our structure, we can form such a gradient by selecting the capacitance of the meta-atoms to exhibit different reflected phases as shown in Fig. 2c. While the surface tuning to implement a phase gradient (according to the generalized law of refraction) has been shown to steer beam anomalously^54^, the presence of sidelobes in the reflected beam weakens the signal in the desired direction. Recently, various works have addressed this shortcoming^55–58^. We have adopted the method in^56^ which finds the surface impedance variation of a lossy metasurface that can redirect the incident beam towards a prescribed direction—note that the coupling to the SIWs renders our proposed metasurface lossy.

**Figure 8:**
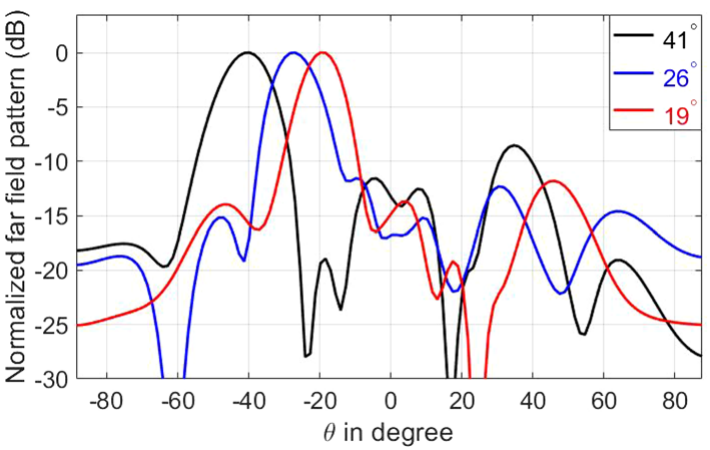
Far-field pattern of three reflected beams redirected from normal incidence. The beam direction is measured with reference to the normal to the RIS.

To illustrate the reconfigurable reflection capability of the proposed hybrid RIS, we design the metasurface masks to redirect a normal incident beam into three different directions, namely $19^{\circ }$, $26^{\circ }$, and $41^{\circ }$ measured from the surface normal. The results of our design are shown in Fig. 8 which verify the ability of the proposed metasurface configuration to redirect reflected patterns (while also offering integrated sensing capabilities). Note that the results in Fig. 8 are reported at 19.1 GHz. This slight frequency shift (from the design frequency 18.9 GHz) can be attributed to quantization errors while selecting varactor capacitances. Another factor contributing to the frequency shift (as well as degrading the overal beam quality) is the fact that we use simulation of infinite periodic elements (Fig. 2a) for selecting capacitive loading of finite array of varying (non-periodic) elements. With application of optimization techniques, one can eliminate such small frequency shifts and improve beam quality^58^. In addition, we can further improve the beam quality by using larger metasurfaces, or by applying the grating suppression technique^59^.

## Discussion

The results presented in the previous sections demonstrate the feasibility of a hybrid RIS with integrated sensing and reflecting capabilities. For this device to be useful in practical settings, several issues need to be addressed in future work. Presented AoA estimation algorithm, while intuitive and useful for demonstration purposes, assumes a single transmitter and cannot easily be extended to multiple transmitters. This is because the considered sensing matrix only includes phase information and ignores half of the information (i.e., amplitude). Furthermore, the process to utilize phase difference is a nonlinear operation. When we have multiple incident waves, their impact is combined coherently following the principle of superposition. As a result, the information for each incident wave is linearly combined with other ones. By using a nonlinear operation on the sampled signal, it becomes complicated to demultiplex the information for the multiple superimposed incident waves. In future works, we plan to use the complex signal received at each sampling waveguide and demonstrate the possibility to detect multiple transmitters (i.e. multiple AoAs). In doing so, we will also update the deduction algorithm based on the rich body of literature on AoA detection methods and use techniques such as MUSIC, or deep learning networks^60,61^.

It is worth noting that the proposed RIS uses mushroom structures as constitutive elements. However, the envisioned operation can be integrated into other structures as well. The primary operational factor determining the type of elements (other than fabrication and cost constraints) is the inter-element coupling and the presence of surface wave modes. If elements are farther from each other, inter-element coupling, and surface waves are mitigated. Such an RIS can enjoy a simple design process, especially for sculpting beams. However, as pointed out in^52^, such structure may suffer from fundamental limitations in their beamforming capabilities unless elements with both electric and magnetic responses are used. On the other hand, the presence of surface wave modes (or other nonlocal modes as discussed in^52^) can in fact be leveraged in generating desired beams. However, we need to take them into account when designing the metasurface masks^56,58^. This process will be pursued in future works. It is worth emphasizing that the role of inter-element coupling and surface wave modes is not limited to beamforming, as they also impact the sensing capabilities. As discussed earlier, we can utilize them to multiplex the information from the wave incident on each meta-atom and reduce the number of sampling circuitry (i.e., receiving RF chains). In other words, when selecting elements for an RIS with integrated sensing capabilities, one needs to balance the trade-offs in beamforming capabilities as well as sampling circuitry complexity.

Another exciting future direction is to tailor the frequency response of the metasurface. As pointed out in^34^, one can use the frequency selectivity of each meta-atom to engineer the (optimum) wideband channel^62^. In such a system, the proposed design can also sense wideband incident beams. On the other hand, while the proposed work only examined the case of single polarized waves, one can envision scenarios that information is multiplexed in the polarization or angular momentum^63,64^. In such cases, depending on the intended applications, we can use polarization independent or polarization sensitive metamaterial elements, such as the geometries proposed in^65–70^.

## Conclusion

In this paper, we proposed a reconfigurable metasurface with integrated reflecting and sensing capabilities, which was designed and investigated via full-wave electromagnetic simulations. As an illustrative example, we showed that the electromagnetic field sensed by the metasurface can be used to infer the AoA of an impinging wave, which constitutes a special case of channel estimation. We also showed the possibility to drastically reduce the number of detectors by leveraging the metasurface’s reconfigurable response. Preliminary results proving that the proposed hybrid metasurface is capable of redirecting the reflected patterns toward the desired directions were also presented. The proposed hybrid metasurface with integrated reflecting/sensing capabilities can find applications in RIS-empowered communication systems, wireless power transfer, and smart sensors.
